# Vitiligo is associated with an increased risk of cardiovascular diseases: a large-scale, propensity-matched, US-based retrospective study

**DOI:** 10.1016/j.ebiom.2024.105423

**Published:** 2024-10-25

**Authors:** Alicja Frączek, Agnieszka Owczarczyk-Saczonek, Ralf J. Ludwig, Gema Hernandez, Sascha Ständer, Diamant Thaci, Henner Zirpel

**Affiliations:** a Dermatology, Sexually Transmitted Diseases and Clinical Immunology, School of Medicine, University Warmia and Mazury in Olsztyn, 10-719, Olsztyn, Poland; b Department of Dermatology, Venerology, Allergology – Section for Inflammatory Diseases, UKSH, Campus Lübeck, Lübeck, Germany; c Lübeck Institute of Experimental Dermatology, University of Lübeck, Lübeck, Germany; d TriNetX, LLC, Cambridge, MA, USA; e Comprehensive Center for Inflammation Medicine, University-Hospital Schleswig-Holstein, Campus Lübeck, Lübeck, Germany

**Keywords:** Vitiligo, Cardiovascular disease, Heart failure, Heart disease, MACE, TriNetX

## Abstract

**Background:**

Vitiligo is an autoimmune disease, characterized by specific destruction of melanocytes. While associations with numerous comorbid conditions, which potentially increase the risk of cardiovascular diseases have been described, data on the risk for cardiovascular disease is inconclusive. To address this relevant knowledge gap, this study aims to identify the risk of cardiovascular disease in vitiligo.

**Methods:**

The US Collaborative Network was accessed using the TriNetX platform, allowing retrospective data retrieval from electronic health records (EHRs) from 57 US based health care organizations (HCOs). Patients with vitiligo and controls were identified by their respective ICD10 codes. Risk of onset of several cardiovascular diseases was determined in patients within 15 years after diagnoses.

**Findings:**

A total of 94 diagnoses with a prevalence of ≥1% in both cohorts, which consisted of 96,581 individuals per group after propensity-score-matching, were identified. Of those, 54 displayed an increased risk in vitiligo. None of the cardiovascular diseases investigated were associated with a decreased risk in patients with vitiligo. Specifically, cerebral infarction occurred in 1.3% of patients with vitiligo, and 1.0% in controls. This difference translated into a hazard ratio (HR) of 1.21 (95% confidence interval [CI] 1.11–1.32, p_adj_ < 0.001). Venous thromboembolism was recorded in 1.34% of cases and 1.02% of controls without vitiligo, resulting in an increased HR of 1.27 (95% CI 1.171–1.38, p_adj_ < 0.001). Further, major adverse cardiovascular events (MACE) as a composite endpoint was evaluated. The risk for MACE was increased following a vitiligo diagnosis (HR 1.28, 95% CI 1.22–1.35, p_adj_ < 0.001), which persisted in both sensitivity analyses.

**Interpretation:**

Patients with vitiligo display an increased risk of onset of cardiovascular diseases as compared to healthy individuals. Thus, vitiligo might require more precise monitoring and systemic treatment.

**Funding:**

This research was supported by the 10.13039/100024172Schleswig-Holstein Excellence-Chair Program from the State of Schleswig Holstein, by the Excellence Cluster *Precision Medicine in Chronic Inflammation* (DFG, EXC 2167), and by 10.13039/501100001659DFG Individual Grant LU 877/25-1.

**Research in context**

**Evidence before this study**

Vitiligo is an autoimmune disorder. The association with cardiovascular diseases has so far not been studied intensively. However, reports on other autoimmune skin diseases indicate an increased risk for cardiovascular diseases. Reported studies are contrasting, indicating either increased risk for several cardiovascular diseases or no increased risk, as for major adverse cardiovascular events (MACE), which is of particular interest due to novel drug options for vitiligo.

**Added value of this study**

Here, a statistically significant increased risk for 54 different cardiovascular diseases was found, which mainly constitute heart conduction disorders, diseases of arteries, arterioles and capillaries, and heart valve diseases. Further, risk for MACE and venous thromboembolism was increased in patients with vitiligo.

**Implications of all the available evidence**

Screening for cardiovascular diseases should be considered during routine monitoring and prophylaxis management of patients with vitiligo. Further, increased risks potentially indicate a systemic inflammatory nature of the disease.

## Introduction

Vitiligo is a chronic, multifactorial skin disease that affects approximately 0.5–2% of the population worldwide.^1^ Characteristic for this condition is the destruction of melanocytes as a result of a complex pathogenesis, including genetic factors, autoimmunity, oxidative stress, and neurochemical mediators.^2^ Despite the persisting social stigmatization, which significantly decreases patients´ quality of life, vitiligo is not only an aesthetic problem manifested by the presence of hypopigmentation lesions.^3^ Instead, association studies indicate that vitiligo, especially its non-segmental form, might be considered as a systemic disease in which the functioning of various organs is impaired.^4^^,^^5^ Moreover, a higher prevalence of comorbidities including thyroid disorders, connective tissue disease, or other dermatological conditions, i.e., psoriasis, atopic dermatitis, and alopecia areata is reported.^6^^,^^7^ Further, vitiligo is also related to insulin resistance, abnormalities in the lipid profile and metabolic syndrome.^8^ Currently, few studies on cardiovascular diseases (CVDs) in individuals with vitiligo exist. These, however, report discrepant results: An increased risk of developing CVDs in vitiligo was reported by Azzazi et al.,^9^ while these results were negated by subsequent studies, demonstrating no higher probability of MACE and cerebrovascular diseases.^10^^,^^11^ A population-based study in Korea indicated a lower risk of mortality, including that resulting from CVDs, among patients with vitiligo.^12^ Despite conflicting results, an emerging association between CVD risk factors and vitiligo was recently concluded in a systematic review.^13^ This is in line with observations made in other chronic inflammatory skin diseases, i.e., psoriasis, hidradenitis suppurativa, systemic lupus erythematosus, prurigo nodularis, cutaneous lupus, and atopic dermatitis.^14^, ^15^, ^16^, ^17^, ^18^ To determine the cardiovascular disease risk in patients with vitiligo, we here performed a retrospective analysis on a large-scale, US-based, electronic health record database.

## Methods

### Study design and database

The study was performed according to established protocols.^15^^,^^17^^,^^19^ Data used for retrospective analysis were retrieved from the US Collaborative Network (USCN) from the federated real-world database TriNetX. HIPAA (Health Insurance Portability and Accountability Act) compliance data were retrieved from electronic health records (EHRs) in bulk format. All data are aggregated, deidentified, and do not contain any data allowing for individual identification. Currently, the USCN consists of 57 health care organizations (HCOs) containing longitudinal EHRs from approximately 96 million patients. Outcome analysis for each identified diagnosis was set to 15 years after index event, where each index event is the first event of the identifying diagnosis required for cohort attribution.

### Ethics

As a federated network, research studies using TriNetX do not require ethical approval. To comply with legal frameworks and ethical guidelines guarding against data re-identification, the identity of participating HCOs and their individual contribution to each dataset are not disclosed. The TriNetX platform only uses aggregated counts and statistical summaries of de-identified information. No Protected Health Information or individualized Personal Data is made available to the users of the platform. Data is protected according to HIPAA regulations.

### Study population and outcome identification

Two cohorts were retrieved from EHRs based on ICD10 codes within the USCN. Patients with vitiligo were identified by ICD10CM: L80. This retrieved 100,047 EHRs with a vitiligo diagnosis before propensity-score-matching (PSM). Control groups were identified by ICD10CM: Z00, excluding those with ICD10CM: L80. Using this inclusion and exclusion criteria, 7,537,768 EHRs were retrieved before PSM. Retrieved numbers are a consequence of available EHRs and are provided in bulk format. To ensure absence of vitiligo in controls, a time constraint of a second diagnosis of Z00 after 1 year or thereafter after first diagnosis, as well as negative selection for presence of ICD10 code L80 was added.

**Supplementary Figure S1:**
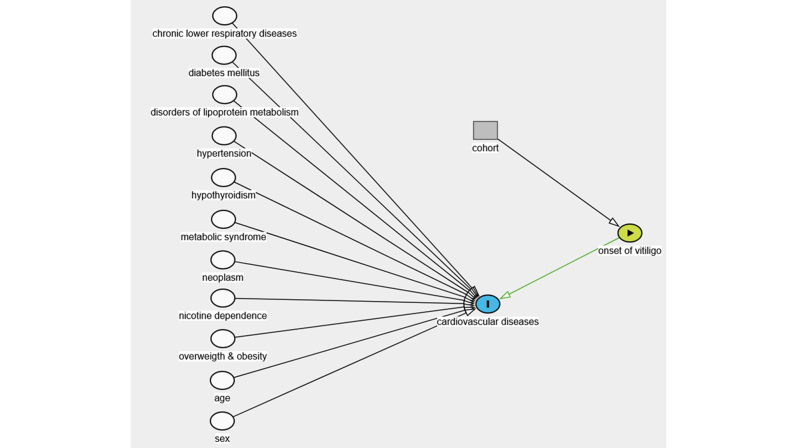


After data retrieval both cohorts were propensity score matched based on the following parameters: Age at index, sex, ethnicity, overweight and obesity (ICD10: E66), diabetes mellitus (ICD10: E08-E13), disorders of lipoprotein metabolism and other lipidemias (ICD10: E78), essential (primary) hypertension (ICD10: I10), chronic lower respiratory diseases (ICD10: J40-J47), hypothyroidism, unspecified (ICD10: E03.9), neoplasm (ICD10: C00-D49), nicotine dependence (ICD10: F17), and personal history of nicotine dependence (Z87.891). Propensity score was estimated using logistic regression, while matching pairs were found by using propensity score density function based on nearest neighbor greedy matching algorithm with a caliper of 0.1 times the standard deviation, with the control group (unexposed group) being matched to the group of individuals with vitiligo (exposed group).^15^^,^^20^^,^^21^ Assumption of logistic regression were met due to large sample sizes, the outcomes being binary outcomes, and PSM variables can be assumed to be independent.^22^ Matching criteria were chosen based on the Framingham risk score, to address potential bias and displayed as cDAG in Supplementary Figure S1.^23^ Of all risk factors defined in the Framingham risk score, family history of ischemic heart disease and other diseases of the circulatory system (Z82.49) was not considered for PSM due to technical limitations of the platform. Z82.49 was chosen to be excluded because of lowest percentage coverage in both cohorts as compared to other risk factors.

To test for false positive association, four independent and unrelated outcomes were selected, and their respective HRs calculated (Supplementary Table S1).

An outcome was defined as a diagnosis of CVDs for each patient within 1 day up to 15 years after the index event. Identification of outcomes after index event was based on ≥1% presence of the CVD, as coded by ICD10, in both cohorts and as identified within the explore outcome function of the TriNetX platform. Patients with a diagnosis of the outcome event prior to the index event were excluded for this particular outcome event analysis. Outcome events identified and based on their respective ICD10 code are displayed in Supplementary Table S2.

For sensitivity analysis two additional analyses were performed in which all analyzed outcomes were included. In the first sensitivity analysis matching was altered by matching exclusively for age and sex, while in the second analysis all matching criteria were included, but the time frame of analysis was shortened to 5 years after the index event (Supplementary Tables S3 and S4).

Time to onset analysis was performed by determining 50% occurrence of outcome event in all individuals with the respective outcome event as provided by anonymized batch data for survival probability and time. Timepoint for start of survival analysis, which in this analysis was equal to origin, was either first diagnosis of vitiligo (ICD10CM: L80) or “encounter for general examination without complaint, suspected or reported diagnosis” (ICD10CM: Z00), with the maximum time of analysis being 15 years after first diagnosis, or first record of the analyzed outcome. The median follow-up time for vitiligo was 1068 days (IQR 2438 days) and the median-follow-up time for control group was 1245 days (IQR 2172). After 15 years, the censoring proportion is 88.90% for the vitiligo cohort and 89.89% for the control cohort. Patients are censored after the last record available in TriNetX.

To ensure sample size meeting minimum required sample size, minimum sample size, as well as width of 95% CI were calculated, resulting in a minimum sample size of 1174 participants, which was met in all analyses (Supplementary Method 1).

### Statistical analysis

Differences between distribution of both cohorts were calculated by log-rank test. For each outcome event the HR with 95% CI was calculated by univariate Cox proportional hazards regression. Proportional hazards test based on scaled Schoenfeld residual was performed. A p value > 0.05 was used to validate the proportional hazards assumption. Kaplan–Meier survival analysis was performed for survival analysis. Pairwise log-rank comparison was run for each disease. Each analysis was performed after propensity score matching. Two-tailed p-values less than 0.05 were considered statistically significant. Bonferroni correction was performed for multiple testing; α (adjust) is indicated in the respective tables.

### Role of funders

The funders had no role in the study design, data collection, analyses, interpretation, or writing the report.

## Results

### Patient demographics

Within the USCN, 100,047 EHRs with vitiligo and 7,537,768 controls were retrieved. Batchwise data retrieval from EHR showed that the average age at index of patients with vitiligo was 38.8 ± 23.3 years, with 54.2% being female and 53.5% being white. Overweight and obesity was recorded in 8.8%, while 6.6% of EHRs indicated nicotine dependence or history of nicotine dependence. Propensity score matching resulted in 96,581 EHRs in both cohorts (Table 1).

**Table 1:** Patient and control demographics and additional risk factors used for propensity score matching based on the Framingham risk score before and after propensity score matching. The propensity-score matching model's covariates are displayed with uncorrected p-values (t-test).

	Before matching				After matching			
	Vitiligo [n = 100,047]	Healthy control [n = 7,537,768]	p value	Standardized difference	Vitiligo [n = 96,581]	Healthy control [n = 96,581]	p value	Standardized difference
Age at index [years]	38.8 ± 23.3	34.6 ± 26.1	<0.001	0.168	38.8 ± 23.3	39.2 ± 23.3	<0.001	0.016
Sex (Female)	54.2% (52,361)	52.6% (3,914,217)	<0.001	0.032	54.2% (52,361)	54.3% (52,458)	0.658	0.007
Ethnicity (Not Hispanic or Latino)	59.9% (57,852)	70.0% (5,206,545)	<0.001	0.213	59.9% (57,852)	59.9% (57,851)	0.996	0.002
White	53.5% (51,655)	60.7% (4,512,868)	<0.001	0.145	53.5% (51,655)	53.5% (51,678)	0.916	0.005
Overweight and obesity	8.8% (8543)	14.2% (1,060,038)	<0.001	0.170	8.8% (8543)	9.3% (9015)	<0.001	0.012
Nicotine dependence	3.7% (3550)	5.9% (440,947)	<0.001	0.105	3.7% (3550)	4.3% (4136)	<0.001	0.007
Personal history of nicotine dependence	2.9% (2781)	5.5% (407,297)	<0.001	0.130	2.9% (2781)	3.2% (3074)	<0.001	0.024
Diabetes mellitus	8.2% (7916)	9.0% (666,792)	<0.001	0.027	8.2% (7916)	8.0% (7739)	0.140	0.003
Essential (primary) hypertension	17.0% (16,378)	24.2% (1,803,967)	<0.001	0.181	17.0% (16,378)	17.1% (16,497)	0.471	0.004
Neoplasms	17.5% (16,379)	18.9% (1,405,611)	<0.001	0.050	17.5% (16,889)	17.4% (16,813)	0.649	0.005
Disorders of lipoprotein metabolism and other lipidemias	16.2% (15,687)	26.7% (1,987,107)	<0.001	0.257	16.2% (15,687)	16.4% (15,879)	0.237	0.006
Chronic lower respiratory diseases	9.8% (9475)	15.3% (1,139,571)	<0.001	0.167	9.8% (9475)	9.9% (9526)	0.697	0.001
Hypothyroidism, unspecified	7.9% (7592)	7.4% (551,284)	<0.001	0.017	7.9% (7592)	7.5% (7288)	0.009	0.007
Metabolic syndrome	0.1% (84)	0.1% (10,089)	<0.001		0.1% (84)	0.1% (95)	0.004	
Family history of ischemic heart diseases and other diseases of the circulatory system	1.8% (1712)	3.6% (175,764)	<0.001		1.8% (1712)	3.0% (2866)	<0.001	

### Increased risk of cardiovascular diseases in patients with vitiligo

The explore outcomes function identified 94 cardiovascular diagnoses that are potentially more frequently observed in vitiligo and were present ≥1% in both cohorts (Table 2). Those diagnoses were assigned by pathogenesis to 10 different cardiovascular groups: cardiomyopathies (4 CVDs), cerebrovascular diseases (7 CVDs), diseases of arteries, arterioles and capillaries (18 CVDs), diseases of veins, lymphatic vessels and lymph nodes (9 CVDs), heart conduction disorders (22 CVDs), heart valve diseases (13 CVDs), heart failure (10 CVDs), ischemic heart diseases (5 CVDs), other forms of heart diseases (3 CVDs), pericardial disorders (2 CVDs), and MACE. Of those 94 CVDs identified, 54 displayed statistically significantly increased risk in patients with vitiligo (p_adj_ < 0.05). For each outcome two sensitivity analyses were performed restricting time of analysis to 5 years and matching exclusively for age and sex (Supplementary Tables S3 and S4). In addition, 4 independent and unrelated outcomes were analyzed, displaying neither significantly increased nor decreased HRs between both cohorts.

**Table 2:** Identified cardiovascular diseases, their respective cardiovascular disease area and their prevalence, hazard ratios, 95% CI, p-values and s values. The Log-rank test was used to compare Kaplan–Meier curve.

Class	Definition	Vitiligo USCN			HC USCN			Hazard ratio	95% Confidence interval		p value (Log rank test)	Adjusted p value after bonferroni correction	S value	Adjusted S value after bonferroni correction
		N of eligible participants	N of outcomes	Risk, %	N of eligible participants	N of outcomes	Risk, %							
Cerebrovascular diseases	Cerebral infarction due to unspecified occlusion or stenosis of cerebral arteries	96,022	494	0.005	96,067	339	0.004	1.43	1.24	1.64	<0.001	<0.001	>13.29	>13.29
	Sequelae of cerebral infarction	96,337	483	0.005	96,349	358	0.004	1.30	1.13	1.49	<0.001	<0.001	>13.29	>13.29
	Cerebral infarction	95,494	1214	0.013	95,621	977	0.010	1.21	1.11	1.31	<0.001	<0.001	>13.29	>13.29
	Other cerebrovascular diseases	95,430	1303	0.014	95,594	1060	0.011	1.19	1.10	1.29	<0.001	<0.001	>13.29	>13.29
	Cerebral infarction, unspecified	95,900	1034	0.011	95,965	867	0.009	1.14	1.05	1.25	0.003	0.282	8.381	1.826
	Occlusion and stenosis of carotid artery	95,783	1059	0.011	95,698	941	0.010	1.09	1.00	1.19	0.048	1	4.381	0
	Occlusion and stenosis of bilateral carotid arteries	96,261	732	0.008	96,183	715	0.007	0.99	0.89	1.09	0.795	1	0.331	0
Diseases of arteries, arterioles and capillaries	Hypertensive chronic kidney disease	95,031	1762	0.019	95,466	1140	0.012	1.52	1.41	1.63	<0.001	<0.001	>13.29	>13.29
	Hypertensive heart and chronic kidney disease	96,188	839	0.009	96,296	558	0.006	1.46	1.31	1.62	<0.001	<0.001	>13.29	>13.29
	Other pulmonary heart diseases	95,648	1136	0.012	95,859	774	0.008	1.45	1.32	1.58	<0.001	<0.001	>13.29	>13.29
	Nevus, non-neoplastic (Applicable to Araneus nevus, Serile nevus, Spider nevus, Stellar nevus)	95,400	1139	0.012	96,023	814	0.008	1.38	1.26	1.51	<0.001	<0.001	>13.29	>13.29
	Hypertensive heart disease with heart failure	96,012	1134	0.012	96,088	813	0.008	1.35	1.24	1.48	<0.001	<0.001	>13.29	>13.29
	Hypertensive heart disease	95,640	1489	0.016	95,777	1077	0.011	1.35	1.25	1.46	<0.001	<0.001	>13.29	>13.29
	Hypotension	94,813	2636	0.028	95,089	1957	0.021	1.32	1.25	1.40	<0.001	<0.001	>13.29	>13.29
	Secondary hypertension	96,112	684	0.007	96,117	506	0.005	1.32	1.17	1.48	<0.001	<0.001	>13.29	>13.29
	Atherosceloritc heart disease of native coronary artery with unspecified angina pectoris	96,376	420	0.004	96,358	320	0.003	1.27	1.09	1.47	0.001	0.094	9.966	3.411
	Atherosceloritc heart disease of native coronary artery without angina pectoris	92,961	2903	0.031	93,532	2285	0.024	1.26	1.19	1.33	<0.001	<0.001	>13.29	>13.29
	Atherosclerosis of native arteries of the extremities	96,179	537	0.006	96,208	420	0.004	1.25	1.10	1.42	0.001	0.094	9.966	3.411
	Atherosclerosis	95,322	1871	0.020	95,398	1564	0.016	1.17	1.09	1.25	<0.001	<0.001	>13.29	>13.29
	Peripheral vascular disease, unspecified	95,536	1272	0.013	95,471	1067	0.011	1.16	1.07	1.26	<0.001	<0.001	>13.29	>13.29
	Athersclerosis of aorta	95,948	1190	0.012	95,943	1002	0.010	1.16	1.06	1.26	0.001	0.094	9.966	3.411
	Hypertensive crisis	96,337	460	0.005	96,355	394	0.004	1.11	0.97	1.27	0.124	1	3.012	0
	Aortic ectasia	96,297	646	0.007	96,288	555	0.006	1.11	0.99	1.25	0.069	1	3.857	0
	Essential (primary) hypertension	76,938	7394	0.096	78,727	7219	0.092	1.07	1.03	1.10	<0.001	<0.001	>13.29	>13.29
	Aortic aneurysm and dissection	96,114	493	0.005	96,120	486	0.005	0.99	0.87	1.12	0.809	1	0.306	0
Diseases of veins, lymphatic vessels and lymph nodes	Acute embolism and thrombosis of unspeficied deep veins of unspecified lower extremity	96,022	513	0.005	96,037	341	0.004	1.47	1.28	1.69	<0.001	<0.001	>13.29	>13.29
	Other venous embolism and throbosis	95,448	1280	0.013	95,479	977	0.010	1.27	1.17	1.38	<0.001	<0.001	>13.29	>13.29
	Pulmonary embolism	95,972	693	0.007	96,038	530	0.006	1.27	1.14	1.43	<0.001	<0.001	>13.29	>13.29
	Lymphedema, not elsewhere classified	96,180	441	0.005	96,237	348	0.004	1.24	1.08	1.42	0.003	0.282	8.381	1.826
	Acute embolism and thrombosis of deep veins of lower extremity	95,820	834	0.009	95,805	680	0.007	1.19	1.07	1.32	0.001	0.094	9.966	3.411
	Venous insufficiency (chronic) (peripheral)	95,587	1155	0.012	95,617	1006	0.011	1.12	1.03	1.22	0.009	0.846	6.796	0.241270431542137
	Varicose veins of lower extremities	95,239	1221	0.013	95,088	1147	0.012	1.04	0.96	1.13	0.368	1	1.442	0
	Asymptomatic varicose veins of lower extremities	95,757	638	0.007	95,649	663	0.007	0.93	0.84	1.04	0.219	1	2.191	0
	Varicose veins of lower extremities with other complications	96,102	632	0.007	95,985	668	0.007	0.91	0.82	1.01	0.084	1	3.573	0
Heart valve diseases	Rheumatic tricuspid insufficiency	96,215	560	0.006	96,244	406	0.004	1.35	1.19	1.53	<0.001	<0.001	>13.29	>13.29
	Other nonrheumatic mitral valve disorders	95,564	657	0.007	95,640	511	0.005	1.27	1.13	1.42	<0.001	<0.001	>13.29	>13.29
	Chronic rheumatic heart diseases	95,546	1461	0.015	95,565	1160	0.012	1.23	1.14	1.33	<0.001	<0.001	>13.29	>13.29
	Nonrheumatic aortic valve disorder, unspecified	95,953	388	0.004	96,008	319	0.003	1.21	1.04	1.40	0.013	1	6.265	0
	Other nonrheumatic aortic valve disorders	95,904	667	0.007	95,996	537	0.006	1.20	1.07	1.34	0.002	0.188	8.966	2.411
	Nonrheumatic aortic (valve) insufficiency	95,779	861	0.009	95,858	723	0.008	1.16	1.05	1.28	0.004	0.376	7.966	1.411
	Nonrheumatic aortic valve disorders	95,460	1465	0.015	95,489	1238	0.013	1.14	1.06	1.23	0.001	0.094	9.966	3.411
	Nonrheumatic mitral (valve) insufficiency	95,024	1723	0.018	95,016	1506	0.016	1.11	1.03	1.19	0.004	0.376	7.966	1.411
	Nonrheumatic mitral valve disorders	94,846	1915	0.020	94,795	1678	0.018	1.11	1.04	1.18	0.003	0.282	8.381	1.826
	Nonrheumatic aortic (valve) stenosis	95,823	669	0.007	95,857	594	0.006	1.10	0.99	1.23	0.086	1	3.540	0
	Nonrheumatic tricuspid valve disorders	95,741	1254	0.013	95,668	1111	0.012	1.08	1.00	1.17	0.066	1	3.921	0
	Nonrheumatic tricuspid (valve) insufficiency	96,113	1135	0.012	96,051	1007	0.010	1.06	0.97	1.15	0.200	1	2.322	0
	Nonrheumatic pulmonary valve disorders	96,163	688	0.007	96,166	632	0.007	1.02	0.92	1.14	0.686	1	0.544	0
Heart failure	Acute on chronic systolic (congestive) heart failure	96,343	397	0.004	96,390	258	0.003	1.48	1.27	1.73	<0.001	<0.001	>13.29	>13.29
	Unspecified diastolic (congestive) heart failure	96,244	736	0.008	96,232	490	0.005	1.46	1.30	1.63	<0.001	<0.001	>13.29	>13.29
	Unspecified systolic (congestive) heart failure	96,320	582	0.006	96,323	390	0.004	1.44	1.26	1.63	<0.001	<0.001	>13.29	>13.29
	Heart failure, unspecified	95,038	1577	0.017	95,289	1109	0.012	1.39	1.29	1.50	<0.001	<0.001	>13.29	>13.29
	Diastolic (congestive) heart failure	95,856	1364	0.014	95,879	957	0.010	1.38	1.27	1.50	<0.001	<0.001	>13.29	>13.29
	Chronic systolic (congestive) heart failure	96,203	685	0.007	96,148	488	0.005	1.35	1.21	1.52	<0.001	<0.001	>13.29	>13.29
	Heart failure	94,516	2301	0.024	94,768	1689	0.018	1.33	1.25	1.42	<0.001	<0.001	>13.29	>13.29
	Combined systolic (congestive) and diastolic (congestive) heart failure	96,277	557	0.006	96,283	406	0.004	1.33	1.17	1.51	<0.001	<0.001	>13.29	>13.29
	Acute on chronic diastolic (congestive) heart failure	96,374	504	0.005	96,396	381	0.004	1.27	1.11	1.45	<0.001	<0.001	>13.29	>13.29
	Systolic (congestive) heart failure	95,949	995	0.010	95,936	767	0.008	1.25	1.14	1.38	<0.001	<0.001	>13.29	>13.29
Heart conduction disorders	Sick sinus syndrome	96,280	359	0.004	96,295	224	0.002	1.58	1.33	1.86	<0.001	<0.001	>13.29	>13.29
	Other specified conduction disorders	96,285	509	0.005	96,321	366	0.004	1.33	1.16	1.52	<0.001	<0.001	>13.29	>13.29
	Left bundle-branch block, unspecified	96,321	369	0.004	96,320	281	0.003	1.27	1.08	1.48	0.003	0.282	8.381	1.826
	Atrioventricular block, first degree	96,157	737	0.008	96,215	559	0.006	1.26	1.13	1.41	<0.001	<0.001	>13.29	>13.29
	Unspecified atrial fibrillation and atrial flutter	94,872	1833	0.019	94,826	1493	0.016	1.20	1.12	1.28	<0.001	<0.001	>13.29	>13.29
	Unspecified atrial fibrillation	94,959	1755	0.018	94,890	1432	0.015	1.20	1.12	1.28	<0.001	<0.001	>13.29	>13.29
	Atrioventricular and left bundle-branch block	95,629	1429	0.015	95,685	1154	0.012	1.20	1.11	1.29	<0.001	<0.001	>13.29	>13.29
	Other specified cardiac arrhythmias	94,865	1531	0.016	94,873	1276	0.013	1.18	1.10	1.27	<0.001	<0.001	>13.29	>13.29
	Ventricular tachycardia	96,201	533	0.006	96,237	441	0.005	1.17	1.03	1.33	0.013	1	6.265	0
	Unspecified atrial flutter	96,227	510	0.005	96,254	423	0.004	1.16	1.02	1.32	0.022	1	5.506	0
	Other conduction disorders	95,459	1655	0.017	95,469	1374	0.014	1.16	1.08	1.25	<0.001	<0.001	>13.29	>13.29
	Atrial fibrillation and flutter	94,679	2001	0.021	94,605	1682	0.018	1.16	1.09	1.24	<0.001	<0.001	>13.29	>13.29
	Unspecified right bundle-branch block	96,016	821	0.009	96,030	691	0.007	1.15	1.04	1.27	0.007	0.658	7.158	0.603
	Other and unspecified right bundle-branch block	95,988	899	0.009	95,994	778	0.008	1.12	1.01	1.23	0.026	1	5.265	0
	Paroxysmal tachycardia	95,668	1285	0.013	95,701	1143	0.012	1.09	1.00	1.18	0.040	1	4.644	0
	Supraventricular tachycardia	96,004	898	0.009	95,984	824	0.009	1.05	0.96	1.15	0.312	1	1.680	0
	Chronic atrial fibrillation	96,323	543	0.006	96,188	502	0.005	1.05	0.93	1.18	0.464	1	1.108	0
	Paroxysmal atrial fibrillation	95,938	1345	0.014	95,708	1240	0.013	1.04	0.97	1.13	0.284	1	1.816	0
	Atrial premature depolarization	96,126	971	0.010	96,068	901	0.009	1.03	0.94	1.13	0.564	1	0.826	0
	Cardiac arrhythmia, unspecified	95,175	1747	0.018	95,080	1687	0.018	1.01	0.94	1.08	0.812	1	0.300	0
	Venticular premature depolarization	95,610	1362	0.014	95,627	1364	0.014	0.96	0.89	1.04	0.288	1	1.796	0
	Persistent atrial fibrilation	96,392	482	0.005	96,349	520	0.005	0.89	0.79	1.01	0.061	1	4.035	0
Cardiomyopathies	Cardiomyopathy, unspecified	96,181	724	0.008	96,072	479	0.005	1.46	1.30	1.64	<0.001	<0.001	>13.29	>13.29
	Other cardiomyopathies	96,041	531	0.006	96,065	355	0.004	1.45	1.27	1.66	<0.001	<0.001	>13.29	>13.29
	Ischemic cardiomyopathy	96,288	386	0.004	96,316	288	0.003	1.30	1.12	1.52	0.001	0.094	9.966	3.411
	Cardiomyopathy	95,653	1082	0.011	95,642	820	0.009	1.28	1.17	1.40	<0.001	<0.001	>13.29	>13.29
Ischemic heart disaeses	Old myocardial infarction	95,708	976	0.010	95,881	689	0.007	1.39	1.26	1.53	<0.001	<0.001	>13.29	>13.29
	Angina pectoris	95,597	968	0.010	95,796	692	0.007	1.38	1.25	1.52	<0.001	<0.001	>13.29	>13.29
	Chronic ischemic heart disease	92,561	3205	0.035	93,213	2561	0.027	1.24	1.18	1.31	<0.001	<0.001	>13.29	>13.29
	Ischemic heart diseases	91,957	3747	0.041	92,767	3014	0.032	1.24	1.18	1.30	<0.001	<0.001	>13.29	>13.29
	Acute myocardial infarction	95,656	1116	0.012	95,847	881	0.009	1.23	1.13	1.35	<0.001	<0.001	>13.29	>13.29
Pericardial disorders	Other diseases of pericardium	96,111	719	0.007	96,237	466	0.005	1.50	1.33	1.68	<0.001	<0.001	>13.29	>13.29
	Pericardial effusion (noninflammatory)	96,324	556	0.006	96,387	387	0.004	1.38	1.21	1.57	<0.001	<0.001	>13.29	>13.29
Other forms of heart disease	Heart disease, unspecified	95,926	850	0.009	96,015	581	0.006	1.44	1.29	1.60	<0.001	<0.001	>13.29	>13.29
	Other ill-defined heart diseases	96,339	584	0.006	96,357	459	0.005	1.22	1.08	1.38	0.001	0.094	9.966	3.411
	Cardiomegaly	94,772	2474	0.026	94,876	1993	0.021	1.20	1.13	1.28	<0.001	<0.001	>13.29	>13.29
MACE	MACE	85,440	4028	0.047	86,002	3042	0.035	1.28	1.22	1.35	<0.001	<0.001	>13.29	>13.29

The top 5 CVD with the highest risk being significantly increased were sick sinus syndrome (HR 1.58, 95% CI = 1.33–1.86, p_adj_ < 0.001), hypertensive chronic kidney disease (HR 1.52, 95% CI = 1.41–1.63, p_adj_ < 0.001), other diseases of pericardium (HR 1.50, 95% CI = 1.33–1.68, p_adj_ < 0.001), acute on chronic systolic (congestive) heart failure (HR 1.48, 95% CI = 1.27–1.73, p_adj_ < 0.001), and acute embolism and thrombosis of unspecified deep veins of unspecified lower extremities (HR = 1.47, 95% CI = 1.28–1.69, p_adj_ < 0.001).

### Increased risk of MACE in patients with vitiligo

Of 85,440 eligible patients with vitiligo 4028 had a diagnosis of MACE after diagnosis, as compared to 3042 individuals in the control group. This resulted in an increased HR of 1.28 (95% CI 1.22–1.35, p_adj_ < 0.001). Observed increased risk persisted in all sensitivity analyses.

### Increased risk of cerebrovascular diseases in patients with vitiligo

**Supplementary Figure S2:**
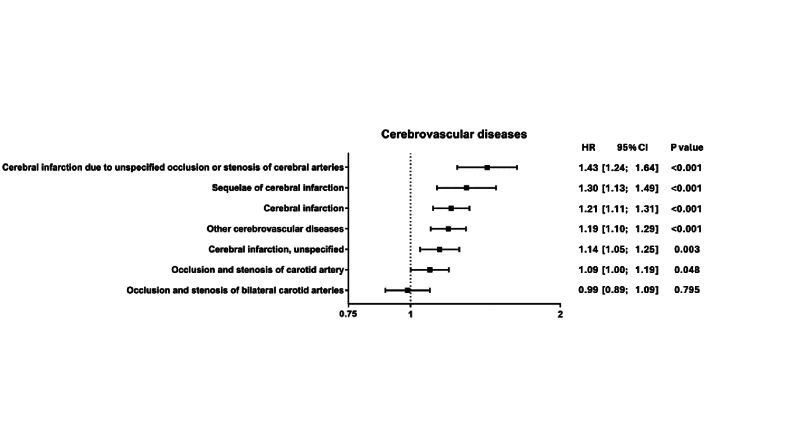


Analysis of cerebrovascular disease displayed highest HR for cerebral infarction due to unspecified occlusion or stenosis of cerebral arteries (HR 1.43, 95% CI 1.24–1.64, p_adj_ < 0.001), followed by sequelae of cerebral infarction (HR 1.30, 95% CI 1.13–1.49, p_adj_ < 0.001) and cerebral infarction (HR 1.21, 95% CI 1.11–1.31, p_adj_ < 0.001) (Supplementary Figure S2).

### Increased risk of diseases of arteries, arterioles, and capillaries in vitiligo patients

**Fig. 1:**
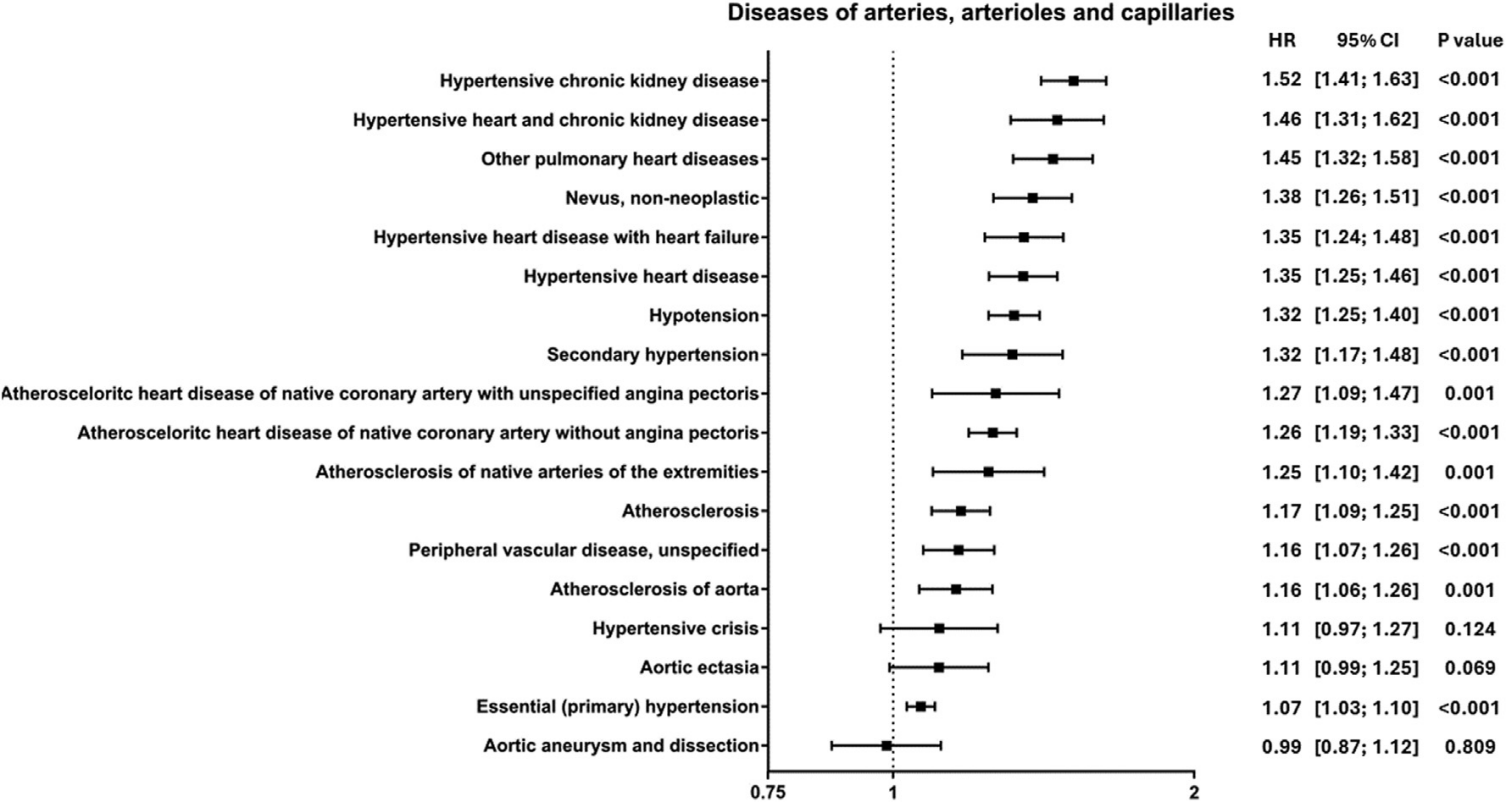
Diseases of arteries, arterioles, and capillaries. Forrest plot displaying HR and 95% CI of identified outcomes of diseases of arteries, arterioles, and capillaries. HR above 1 indicates an increased risk in patients with vitiligo, while a HR below 1 indicates a decreased risk in patients with vitiligo. Outcomes are listed by descending order of their HR.

Of diseases affecting arteries, arterioles, and capillaries hypertensive chronic kidney disease displayed highest HR (HR 1.52, 95% CI 1.41–1.63, p_adj_ < 0.001), followed by hypertensive heart and chronic kidney disease (HR 1.46, 95% CI 1.31–1.62, p_adj_ < 0.001) and other pulmonary heart diseases (HR 1.45, 95% CI 1.32–1.58, p_adj_ < 0.001). Further, hypertensive heart disease (HR 1.35, 95% CI 1.25–1.46, p_adj_ < 0.001), hypotension (HR 1.32, 95% CI 1.25–1.40, p_adj_ < 0.001), and atherosclerosis (HR 1.17, 95% CI 1.09–1.25, p_adj_ < 0.001) were slightly increased. Aortic aneurysm and dissection were the only diagnosis to be found being decreased, however, not to a significant level (HR 0.99, 95% CI 0.87–1.12, p_adj_ = 1) (Fig. 1).

### Increased risk of heart valve diseases in patients with vitiligo

**Supplementary Figure S3:**
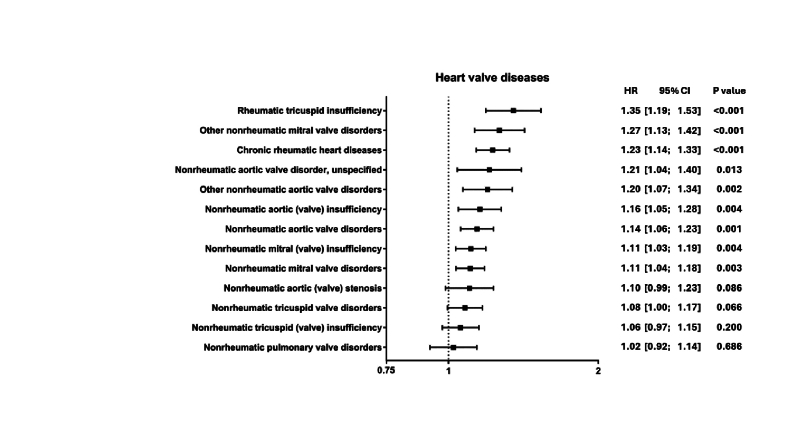


Within the group of heart valve diseases, the diagnosis rheumatic tricuspid insufficiency displayed the highest HR (HR 1.35, 95% CI 1.19–1.53, p_adj_ < 0.001). Of the remaining 12 identified diagnoses 11 were nonrheumatic disorders/insufficiencies with other nonrheumatic mitral valve disorders showing the highest HR (HR 1.27, 95% CI 1.13–1.42, p_adj_ < 0.001), while 4 diagnoses were not significantly altered. The third highest HR was by chronic rheumatic heart diseases (HR 1.23, 95% CI 1.14–1.33, p_adj_ < 0.001) (Supplementary Figure S3).

### Increased risk of heart conduction disorders in patients with vitiligo

**Fig. 2:**
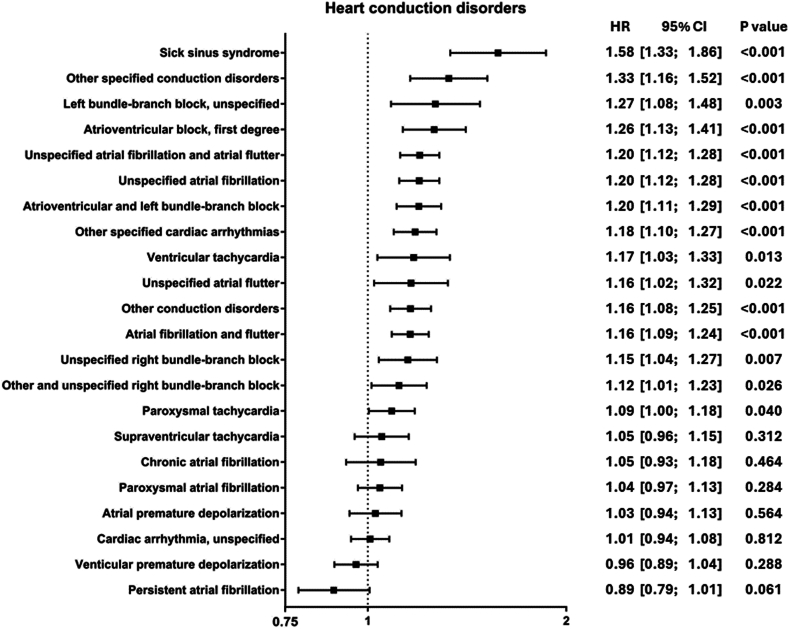
Heart conduction disorders. Forrest plot displaying HR and 95% CI of identified outcomes of heart conduction disorders. HR above 1 indicates an increased risk in patients with vitiligo, while a HR below 1 indicates a decreased risk in patients with vitiligo. Outcomes are listed by descending order of their HR.

The category with the most diagnoses identified (a total of 22) was heart conduction disorders. In here, sick sinus syndrome (HR 1.58, 1.33–1.86, p_adj_ < 0.001), other specified conduction disorders (HR 1.33, 95% CI 1.16–1.52, p_adj_ < 0.001), and left bundle-branch block, unspecified (HR 1.27, 95% CI 1.08–1.48, p_adj_ = 0.282) displayed highest HRs. Three additional bundle-branch block diagnoses, 6 diagnoses on fibrillation and flutter, and three diagnoses on tachycardia were identified. Two diagnoses displayed a decreased HR: ventricular premature depolarization (HR 0.96, 95% CI 0.89–1.04, p_adj_ = 1) and persistent atrial fibrillation (HR 0.89, 95% CI 0.79–1.01, p_adj_ = 1). However, HR of both diagnoses were not altered significantly (Fig. 2).

### Increased risk of other cardiovascular diseases in patients with vitiligo

**Supplementary Figure S4:**
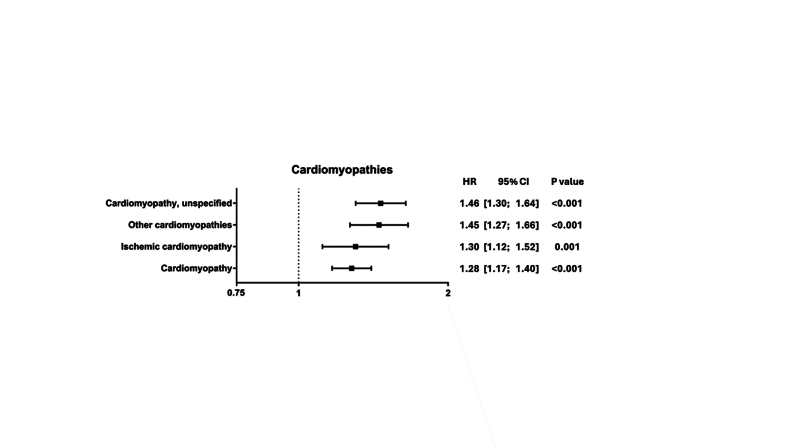


**Supplementary Figure S5:**
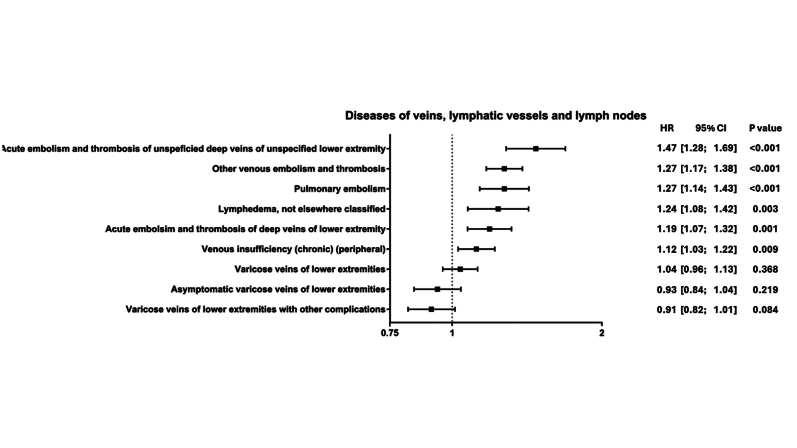


**Supplementary Figure S6:**
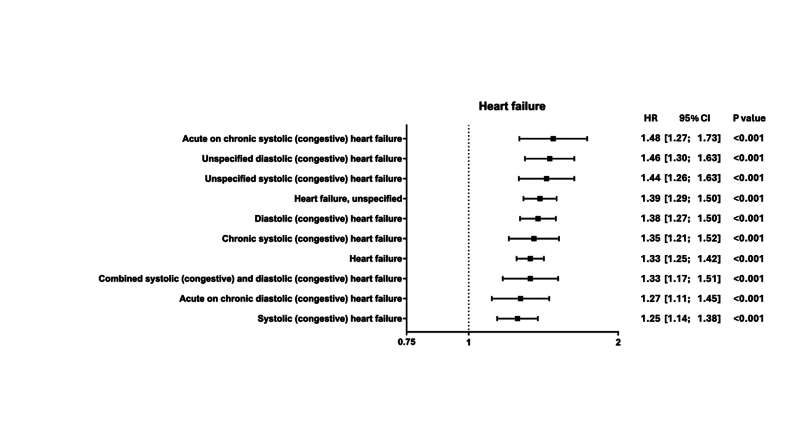


**Supplementary Figure S7:**
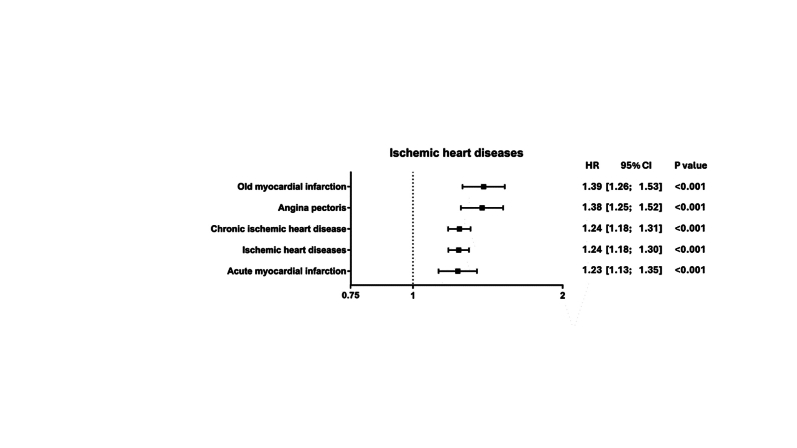


**Supplementary Figure S8:**
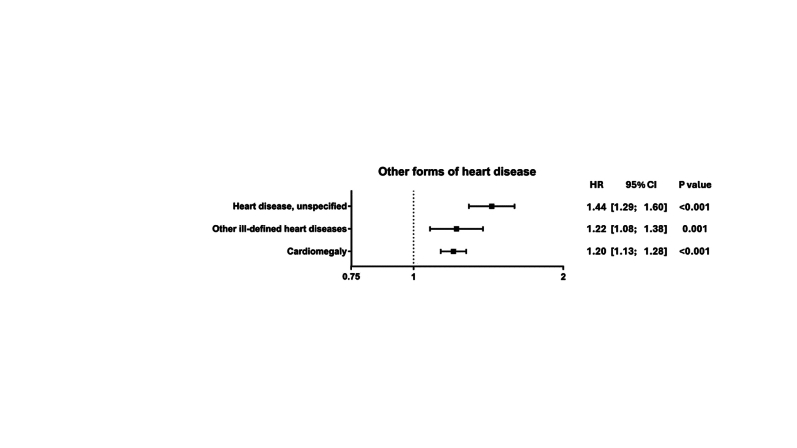


**Supplementary Figure S9:**
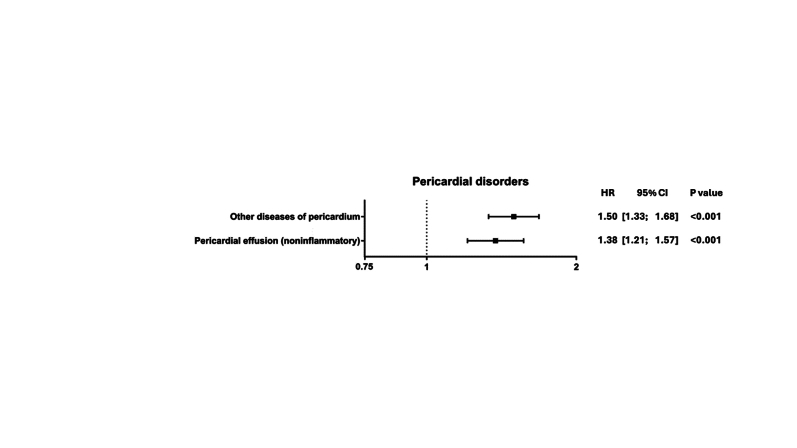


A total of 24 other cardiovascular diagnoses within the remaining 6 cardiovascular disease groups were identified, including heart failure (HR 1.33, 95% CI 1.25–1.42, p_adj_ < 0.001), cardiomyopathy (HR 1.28, 95% CI 1.17–1.40, p_adj_ < 0.001), ischemic heart diseases (HR 1.24, 95% CI 1.18–1.30, p_adj_ < 0.001), angina pectoris (HR 1.38, 95% CI 1.25–1.52, p_adj_ < 0.001), old myocardial infarction (HR 1.39, 95% CI 1.26–1.53, p_adj_ < 0.001), and acute myocardial infarction (HR 1.23, 95% CI 1.13–1.35, p_adj_ < 0.001) (Supplementary Figures S4–S9).

### Earlier onset of cardiovascular disease in patients with vitiligo

To determine if time to onset after the index event differed, timepoints of the 50% survival probability of the respective outcome events were calculated. The 5 diagnoses with the highest HRs, as well as the diagnoses described in the section titled other cardiovascular diseases were analyzed. Overall diagnoses were made 2.95 (±0.72) years earlier in patients with vitiligo as compared to controls. The average year to diagnosis was 7.92 (±0.34) and 10.77 (±1.06) years in individuals with vitiligo and controls, respectively. Sick sinus syndrome, which displayed the highest HR had a time to onset of 7.49 years in patients with vitiligo, as compared to 12.96 years in controls (Table 3).

**Table 3:** Time to onset of cardiovascular diagnoses in patients with vitiligo and controls.

Diagnosis	Vitiligo	Control	Vitiligo	Control
	[d]	[d]	[y]	[y]
Acute embolism and thrombosis of unspecified deep veins of unspecified lower extremities	2734	4402	7.49	12.06
Acute myocardial infarction	2999	3466	8.22	9.50
Acute or chronic systolic (congestive) heart failure	3116	4140	8.54	11.34
Angina pectoris	2785	3977	7.63	10.90
Cardiomyopathy	2912	3459	7.98	9.48
Heart failure	3017	3874	8.27	10.61
Hypertensive chronic kidney disease	2904	4525	7.96	12.40
Ischemic heart diseases	2727	3290	7.47	9.01
Old myocardial infarction	2790	3670	7.64	10.05
Other diseases of pericardium	2670	3700	7.32	10.14
Sick sinus syndrome	2734	4732	7.49	12.96

Columns two (patients with vitiligo) and three (controls) display the time to onset of each diagnosis in days [d], while columns four (patients with vitiligo) and five (controls) display the time of onset of each diagnosis in years [y].

## Discussion

In this retrospective study based on data from EHRs within the USCN of TriNetX, 94 cardiovascular diagnoses were identified being present with a prevalence of ≥1% in patients with vitiligo. Of those, 54 displayed an increased risk in individuals with vitiligo as opposed to patients without vitiligo, with the remaining diagnoses not displaying any significantly increased risk. Presented results indicate that patients with vitiligo demonstrate an increased risk of developing cardiovascular diseases such as cerebral infarction, acute myocardial infarction, pulmonary embolism, and venous thrombosis, while showing that the overall risk of MACE is higher. These findings are contrary to data obtained from another health insurance database describing decreased risk for several comorbidities, including cerebrovascular diseases, in patients with vitiligo as compared to controls and to findings from a Korean population-based cohort study, in which lower risk of mortality as consequence of CVDs was found.^11^^,^^12^ While both studies are based on health insurance data, too, they focus on Korean population as compared to the US population in this study. Further, this study particularly focused on CVDs, thus matching included known risk factors, which were absent in both other studies, potentially explaining observed differences.

Venous thromboembolism (VTE) is reported to be the third most common vascular disease, after myocardial infarction and stroke.^24^ In comparison to the control group, an increased risk of thromboembolism was found, in particular acute embolism and thrombosis of unspecified deep veins of unspecified lower extremity. Further, here reported results suggest a higher prevalence of pulmonary embolism within patients with vitiligo. In contrast, a previously published study, also encompassing electronic health care data in a retrospective format, found no altered risk of developing VTE with deep vein thrombosis (DVT) and pulmonary embolism (PE) in vitiligo.^25^ The discrepancy between both studies might be explained by the differences in cohort sizes, as this study included 12 times more individuals with vitiligo and by matching, since here Framingham risk factors were chosen for matching, while the study by Schneeweiss et al. used matching factors for VTE, which in this study had been analyzed outcomes.

Here, an increased risk of ischemic heart diseases including angina pectoris and acute and old myocardial infarction in vitiligo was found. This is consistent with findings of a study conducted by Tang et al., where among patients with vitiligo with an age ≥60 years old or with a BMI≥ 24 kg/m^2^ symptoms of coronary heart disease were more frequently reported than in individuals without vitiligo. However, no similar association was found in patients with vitiligo aged 40–59 years or non-overweight.^26^ A complementary relationship was also noticed among patients with SLE, where the development of coronary artery diseases was more frequent than in controls.^27^

Additionally, the presented analysis indicated positive correlation with heart failure within individuals with vitiligo. So far, similar observations have been published in cutaneous lupus erythematosus (CLE), discoid lupus erythematosus (DLE), and bullous pemphigoid (BP),^17^^,^^28^, ^29^, ^30^ however, no such association was found in patients with psoriasis.^31^ To the best of the authors’ knowledge, there have been no reports describing the risk of developing heart failure in vitiligo, therefore, that publication is the first to indicate such complication among patients.

Highest identified HR (1.58) of a cardiovascular disease in this study was sick sinus syndrome. To the best of the authors' knowledge no association studies between vitiligo and sick sinus syndrome have so far been reported. However, onset was described in a single case of a patient previously diagnosed with vitiligo and Hashimoto's thyroiditis.^32^ Further, sick sinus syndrome was associated with common cardiovascular risk factors, but also with inflammatory auto-immune diseases, such as diabetes.^33^ Moreover, the analysis indicates an increased risk of developing bundle-branch and atrioventricular blocks. In turn, presented data indicate no correlation with aortic aneurysm and dissection, atrial and ventricular premature depolarization. None of these diseases had so far been addressed in vitiligo.

Obtained results indicate that individuals with vitiligo are at increased risk of developing atherosclerosis, which can lead to the development of further cardiovascular diseases. Similar findings were reported by Azzazi et al., where a higher frequency of diagnosed atherosclerotic plaques and increased common carotid intima media thickness (CIMT) among patients with vitiligo was found.^9^ However, no significant association with disease activity was reported. Contrary, data demonstrated by Namazi et al. showed a positive correlation between severity and disease duration with subclinical atherosclerosis development. Furthermore, evaluated mean intima-media thickness of the common carotid artery (MIMT-CCA) as an index of subclinical atherosclerosis displayed increases in individuals with vitiligo, however, not statistically significant.^34^

Even though both cohorts were matched for hypertension, several hypertensive diseases were identified being significantly increased in vitiligo. This study's findings indicate a higher prevalence of hypertensive chronic kidney disease, which was the complication with the second highest HR among patients with vitiligo as compared to the control group. This outcome is in line with results of a retrospective case–control study with 122 participants, where authors pointed towards little distinction in the risk of developing hypertension between patients with vitiligo and the control group; however, a significant correlation with kidney diseases was demonstrated.^35^

In other inflammatory diseases, the increased risk for cardiovascular disease mainly stemmed from certain groups of patients—with disease severity as a significant driver of cardiovascular disease risk.^18^^,^^36^^,^^37^ Hence, the here observed increased CVD risks potentially stem from patients with a high disease severity, which could not be analyzed in this study due to absence of severity scores in the platform.^38^ In addition, potential influence of other comorbidities might impact on the development of CVDs, which is impacting epidemiological studies on vitiligo in general. Further, analysis of respective forms including segmental, non-segmental or mixed vitiligo, which might be of different pathophysiology, was not possible due to lack of ICD10CM codes for certain vitiligo subtypes.

The nature of our study pertains from drawing any causal relationships between vitiligo and cardiovascular disease risk.

To address causality Mendelian randomization or prospective studies can be performed, as was seen in other chronic inflammatory skin diseases. Particularly, IL-17 driven inflammation in psoriasis has been identified as a causal link for CVDs.^36^^,^^39^^,^^40^ In vitiligo, several causative options are possible: First, the inflammatory nature of subtypes of vitiligo might result in systemic inflammation causing i.e., atherosclerotic plaque formation, whereby increased immune activation was reported for non-segmental, but remains elusive for segmental vitiligo.^5^^,^^41^^,^^42^ Further, disease activity and severity might play a crucial role in the development of several CVDs.^5^ Lastly, therapeutic options such as cyclosporin or oral steroids can cause dyslipidemia or hypertension, promoting development of CVDs, on the one hand, while UVB 311 phototherapy may reduce the risk of both cardiovascular and cerebrovascular events, which may have an impact on the obtained results.^43^, ^44^, ^45^

### Limitations

This study has several limitations which need to be mentioned. First, its retrospective design is based on data retrieval from EHRs. Second, coded diagnoses within EHRs can be prone to errors, allowing potential misdiagnosis or unprecise reporting. Third, data obtained from the TriNetX network do not include clinical scores, which pertain to investigations relating to the risk of CVD in different vitiligo presentations of distinct clinical presentations. Fourth, disease duration, applied form of treatment, as well as severity, activity and subtype of vitiligo are not available in detail. Fifth, analyzed outcomes with low total number must be interpreted with caution. However, this was accounted for by excluding diagnoses with appearance below 1% in investigated cohorts. Sixth, this study reports HR calculated over the time of 15 years, which might result in time dependent changes in HR. Further, the built-in selection bias in HR must be considered, given that individuals more prone to develop CVDs might disease earlier and thus, the group of prone individuals in the control group increase over time.^46^ Seventh, although some findings demonstrated confidence intervals above 1, they were not statistically significant after Bonferroni correction. This suggests that while the results show a potential pattern of association, the strength of evidence weakened when accounting for multiple comparisons. Given the exploratory nature of the study and the conservative nature of the Bonferroni correction, there is a possibility that true associations may have been missed, warranting further investigation with more targeted hypotheses. Eighth, data is protected according to HIPAA regulations and individualized data is not available, which results in limitations for data analysis, such as reporting variables with skewed distribution. Finally, risk factors and confounders were chosen based on previous literature, while stratification and multivariable regression for confounder identification were not possible. due to data protection.

In conclusion, this large-scale study indicates that patients with vitiligo seem to be at an increased risk for the development of cardiovascular diseases, which may give clinicians beneficial insights for patient monitoring and prophylaxis management. The causal relationship between vitiligo and cardiovascular disease needs to be established in prospective clinical investigations.

## Contributors

AF and HZ conceptualized the study. AOS, RJL and DT acquired funding. AF, RJL, SS, and HZ accessed and verified the data. AF, RJL, SS, GH and HZ analyzed the data. AF and HZ drafted the manuscript. AOS, RJL, SS, GH, DT, and HZ revised the manuscript. All authors read and approved the final version.

## Data sharing statement

The data that support the findings of this study are available from the corresponding author, HZ, upon reasonable request.

## Declaration of interests

AO-S has received honoraria or fees for serving on advisory boards, as a speaker, as a consultant from AbbVie, Allergan, Almirall, Janssen, Leo Pharma, Lilly, Medac, Novartis, Sandoz, Sanofi, Sunpharm.

RJL has received honoraria for speaking or consulting or has obtained research grants from Monasterium Laboratories, Novartis, Lilly, Bayer, Dompe, Synthon, Argen-X, and Incyte during the last 3 years.

GH is an employee of TriNetX.

SS has received honoraria for speaking or consulting from AbbVie, Bristol-Myers Squibb, Janssen-Cilag, Pfizer and UCB.

DT has received honoraria or fees for serving on advisory boards, as a speaker, as a consultant from AbbVie, Amgen, Almirall, Bristol-Meiers-Squibb, Boehringer Ingelheim, Celltrion, Galderma, Leo Pharma, Lilly, New Bridge, Novartis, Janssen-Cilag, Pfizer, Regeneron, Sanofi, l, Sun Pharmaceuticals, and UCB and grants from AbbVie, Leo Pharma and Novartis.

HZ has received support for attending meetings and/or travel from Pfizer, UCB Pharma, Almirall, Janssen, TriNetX.

All other authors declare no conflict of interest.

## Appendix A Supplementary data

Supplementary methods

Supplementary Table S1 Diseases and procedures unrelated to vitiligo or cardiovascular diseases and their prevalence, hazard ratios, 95% CI, and p-values (Log-rank test).

Supplementary Table S2 Identified cardiovascular diseases with a prevalence ≥1% and their respective ICD10CM codes.

Supplementary Table S3 Sensitivity analysis of identified cardiovascular diseases, their respective cardiovascular disease area and their prevalence, hazard ratios, 95% CI and p-values (Log-rank test), when matching only for age and sex.

Supplementary Table S4 Sensitivity analysis of identified cardiovascular diseases, their respective cardiovascular disease area and their prevalence, hazard ratios, 95% CI and p-values (Log-rank test), when restricting time of analysis to 5 years.
