# Supplementary material for: Vitiligo is associated with an increased risk of cardiovascular diseases: a large-scale, propensity-matched, US-based retrospective study
Source: eBioMedicine. 2024 Oct 25;109:105423. doi: 10.1016/j.ebiom.2024.105423 (PMC11543909; doi:10.1016/j.ebiom.2024.105423)
Supplement: Supplementary methods [file mmc1.docx]

Supplementary methods:

Calculation of the width to estimate required group sizes

To evaluate whether the sample sizes of the two independent cohorts (group 1 and group 2) in this study are adequate based on the precision of the estimates, the 95% confidence intervals for the difference in proportions were calculated. The Width of the CI should be no greater than 0.01. First the individual proportions were calculated based on the smallest available percentages per outcome before analyses (group 1: p1 = 362/86,558 ≈ 0.00418; group 2: p2 = 302/86,453 ≈ 0.00349). Next, the standard error (SE) of the difference in proportions (p1-p2) was calculated according to SE = √((p1(1 - p1)/n1) + (p2(1 - p2)/n2)), where n1 = 86,558 (total sample size for group 1) and n2 = 86,453 (total sample size for group 2), resulting in SE ≈ 0.000297. Third, the 95% CI for the differences in proportions were calculated by (p̂1- p̂2) ±1.96 x SE, where p̂1 and p̂2 are the sample proportions for group 1 and group 2, respectively. This resulted in the 95% CI being 0.00069 ± 0.000582, with the lower bound = 0.000108 and the upper bound = 0.001272. Lastly, the Width of the Confidence Interval was estimated resulting in the width of the 95% CI being 0.001164. In conclusion the Width of the 95% Confidence Interval for the difference in proportions is approximately 0.001164, which is much less than the desired width of 0.01. This indicates that the sample sizes of 86,558 for group 1 and 86,453 for group 2 are more than adequate to achieve the required precision for the estimates.

Calculation of the minimum required sample size

To determine the required minimum sample size for each cohort to achieve a width of 0.01 for the 95% confidence interval of the difference in proportions, the confidence interval width was calculated according to Width = 2 x Z x SE with Z being the Z-score for the desired confidence level (for 95%, Z ≈ 1.96) and SE being the standard error of the difference in proportions, resulting in SE = 0.01/(2x1.96) ≈ 0.00255. Second, to relate SE to the sample sizes, the standard error for the difference in proportions is given by: SE = √((p1(1 - p1)/n1) + (p2(1 - p2)/n2)). Since both cohorts are matched equal sample sizes was assured (n1 = n2 = n) and the observed proportions p1 ~ 0.00418 and p2 ~ 0.00349 received, resulting in n = 0.00763 / 0.0000065025 ≈ 1,173.35. In conclusion, to achieve a 95% confidence interval width of 0.01 for the difference in proportions, the required minimum sample size for each group is approximately 1,174. Therefore, both groups should have at least 1,174 participants to ensure the desired precision in the estimates, which was met by minimum numbers of patients being 86,453.
